# Embracing Real AI: A call to action for medical physicists in healthcare

**DOI:** 10.1002/acm2.14456

**Published:** 2024-07-18

**Authors:** Dee H. Wu, Olga Pen, Yi Wang, Robin Stern, J. Daniel Bourland, Mahadevappa Mahesh

**Affiliations:** ^1^ Section of Medical Physics Dept. of Radiological Sciences University of Oklahoma Health Sciences Center / OU Health Oklahoma City Oklahoma USA; ^2^ Dept. of Radiation Oncology Atrium Health Charlotte North Carolina USA; ^3^ Dept. of Radiation Oncology Massachusetts General Hospital, Harvard Medical School Boston Massachusetts USA; ^4^ Dept. of Radiation Oncology University of California Lodi California USA; ^5^ Dept. of Radiation Oncology School of Medicine Medical Center Blvd Dept Radiation Oncology Wake Forest University Winston‐Salem North Carolina USA; ^6^ Depts. of Radiology and Medicine Johns Hopkins University Baltimore Maryland USA

## QUICK GLANCE

The article “Embracing Real AI: A Call to Action for Medical Physicists in Healthcare” urges medical physicists to prepare for the integration of artificial intelligence (AI) into healthcare practices, emphasizing their pivotal role in adapting to technological advancements. The authors advocate for embracing AI through advocacy, broadening perspectives, and enhancing coordination and communication. They propose an ABC strategy focusing on increasing educational initiatives, fostering interdisciplinary collaboration, and creating team collaboration to facilitate AI integration. The commentary highlights AI's potential in enhancing diagnostics, personalizing medicine, and automating routine tasks while addressing challenges such as data sharing and the role of federated learning. The article calls for medical physicists to lead in embracing AI, emphasizing continuous learning and collaboration to leverage its potential for improving healthcare and patient care.

## 1 INTRODUCTION

Medical physicists have consistently demonstrated strong interest in developing proficiency in the adoption of new technological advancements. The roots of the profession come from the radiation sciences, including radiation protection, radiation therapy, diagnostic imaging, and nuclear medicine.^1^ As science and technology continued to evolve, medical physicists' roles have extended into other non‐radiation domains, such as non‐ionizing‐radiation‐based imaging (ultrasound and magnetic resonance), molecular imaging, computer aided diagnosis (CAD), information technologies, and data science.^2^

In addition, medical physicists gradually have adopted increasingly more active roles in ensuring the professional education of other radiology/radiation oncology team members, maintaining high quality standards via quality assurance (QA) methods. They also play a major role in advising the hospital management on medical devices and software acquisition. The continuing expansion of these roles and responsibilities has put medical physicists on the forefront of embracing emerging technologies, making the profession one of the most technical and versatile in healthcare settings. Currently, as our field grows in importance, we medical physicists seek to continue to engage in significant ways to for increased contributions and roles in human health.

This commentary/opinion urges medical physicists to prepare for their expanding roles in the field of AI and its implementation and oversight in clinical practice. Medical physicists must embrace “Real AI” to help integrate AI into healthcare practices. Conceptually we advocate for a strategy that involves Real AI through advocacy, broadening, and enhancing coordination/communication (an ABC strategy). In our current and future work medical physicists will use AI to automate routine tasks, allowing medical physicists to focus on more complex tasks. Furthermore, Medical Physics will use AI to enhance efficiency, safety, diagnostic and therapeutic applications, and for personalized medicine. However, as we have done in the past with other complex concepts (such as radiation), medical physicists need to be prepared for the potential risks and ethical dilemmas associated with AI, such as bias and lack of transparency. It will be important that Medical Physicists prepare for the rapidly changing AI landscape, and continue learning, gain hands‐on experience, and collaborate with other AI experts in the healthcare environment.

### 1.1 AI in healthcare: Implementation, management, and ethical considerations for medical physicists

This paper aligns with the already approved guidance document developed by the AAPM in conjunction with International Atomic Energy Agency (IAEA)^3^ that discusses how medical physicists can ensure the effective implementation and management of AI systems. It is crucial for the Clinical Quality Management Program (CQMP) personnel to receive regular training and updates on relevant guidelines and legislation. Clear communication channels should be established with IT experts, vendors, and other stakeholders for smooth coordination.^4^ Comprehensive documentation should be developed to ensure compliance with contractual obligations and guidelines. The clinical team should be involved in acceptance testing and discussions, depending on the clinical purpose of the AI system.^4^ Protocols for data collection and curation should be established, along with the development of standardized validation datasets for performance evaluation.^4^ A system for monitoring updates to AI systems and models should be implemented, with the CQMP leading new acceptance/commissioning rounds for any updates. Lastly, mechanisms for continuous evaluation and improvement of the CQMP processes should be established, which could involve regular audits, feedback mechanisms from end‐users, and incorporating lessons learned from previous rounds.^4^

Nowadays, major healthcare systems in the US consider their data as immensely valuable assets that require rigorous protection to ensure Health Insurance Portability and Accountability Act (HIPAA) compliance, as well as intellectual property considerations. It can be very difficult for researchers to share clinical data with vendors for development purposes without a significant return being specified to the institution, such as joint intellectual property or substantial grant funding. Instead, these healthcare systems encourage their researchers to commercialize their findings independently, allowing the institution to retain full rights to intellectual property. That said, the realization of federated learning would be a significant advancement. To achieve this, a powerful pre‐trained model that would be adaptable to operation on different scales and in various clinical scenarios is necessary. It is plausible that local adaptation may not require substantial computing power or AI expertise. This concept is particularly intriguing and could be beneficial to smaller centers and clinics in underserved areas. However, the primary challenge is the cost. As we become more reliant on AI systems like OpenAI's ChatGPT or Google Gemini, we often overlook the fact that these conveniences come with a hefty price tag, costing billions of dollars to develop and maintain.^5^

As medical physicists we and other healthcare professionals can anticipate that AI will significantly transform healthcare, improving efficiency, accuracy, and the level of detail that can be extracted from imaging, and methods of therapy. These technological advancements are expected to bring immense value to the field, offering a new horizon in diagnostic and therapeutic capabilities. Yet, we also must recognize that it also introduces potential significant risks and ethical dilemmas. One of the primary concerns is the possibility of bias in AI, which can stem from the training data, the algorithms, or their application, leading to potentially detrimental effects on patient care. As medical physicists, we should acknowledge that the complexity and lack of transparency in AI decision‐making processes present obstacles in terms of accountability and rectifying errors and requires greater oversight and responsibility. The integration of AI also has great capacity in redefining the role of medical physicists, impacting education and employment within the field. Addressing these issues necessitates the creation of ethical standards for AI in healthcare, emphasizing transparency, responsibility, and equity, with contributions from diverse stakeholders, including patients, medical professionals, and ethicists.^6^ Such measures are crucial to ensure the responsible utilization of AI in healthcare, and ultimately serve the best interests of patients and society. We anticipate that continued guidance from our professional societies will be helpful as our collective communities develop methods and approaches that help us learn, adopt, and employ AI responsibly.

### 1.2 The ABC and Real AI plan

Through an ABC strategy, medical physicists will position themselves into a pivotal role to play in leading the integration of AI into healthcare. The AAPM Ad Hoc Advisory Committee on AI Boot Camps (AHAIBC) is developing the concept of Real AI as a way to quickly express the ABCs of how to prepare that could be salient and memorable. The ABC concept and process involves several key steps, including advocacy, broadening perspectives, and improving coordination and communication. Building on past campaigns by the AAPM we have initiated our approach to Real AI through an ABC strategy modeled on one AAPM acronym for strategic planning that includes:

1. Advocacy: increase educational initiative, public awareness, and recommending processes at all levels of the clinical workforce, as well as patient engagement.
2. Broadening Perspectives: encourage Interdisciplinary Collaborations that allow medical physicists to work with professionals from other disciplines such as computer science, data science, and biomedical engineering, to gain insights into different perspectives on AI applications in healthcare. This enables medical physicists to provide continuing education and connect the community with research opportunities.
3. Improving Coordination and Communication through creating team collaboration: enhance communication with healthcare professionals, administrators, and patients by clearly defining and articulating the role of medical physicists in AI applications. Promote the sharing of knowledge, as exemplified by creating data repositories through contributions, to further creating the foundation of our understanding and application of AI in the field.

We consider the concept of Real AI in our context to be aimed at providing and/or qualifying a ready AI product that has undergone a rigorous QA process, that is free of false additives and biases, with data carefully curated to represent the demographics and be attuned to the needs of the clinic, sourced with proper ingredients, and abiding by laws and regulations that can ensure the product serves the common health needs of patients and benefits the public's interest.

What AI ‘is’ and what it ‘is not’ is a complex topic that warrants further exploration and understanding, but one vital for comprehension of what utility AI can fulfill in the clinical process, what its advantages and limitations are, and how it can be curated to perform in the clinical scenarios relevant to a particular radiology/radiation oncology practice. Multiple data‐analysis algorithms have been created over the course of years, and not all of them qualify as AI.^7^ What distinction(s) lie in what constitutes AI? One possible interpretation is that AI is a system that can adapt to new data, or a system that generates insights driven by data. AI systems are designed to “learn” and adapt to new data and be stable over the course of introducing data perturbations or employ model adaptation mechanisms. AI systems can adjust the underlying data‐processing mechanisms based on the input they receive, which allows them to improve their performance and make more accurate predictions or decisions over time. This is often achieved through techniques such as machine learning, where algorithms are trained on a dataset and then used to make predictions or decisions without being explicitly programed to perform the task.^8^ Understanding how such datasets are selected, what data needs to be fed into AI model to achieve desired results, and how to prevent common pitfalls and ethical conundrums associated with the use of AI models requires additional training that might yet be lacking in the traditional training of the radiology/radiation oncology adjacent specialists.

The scope of involvement of each member of the team when it comes to AI integration into the clinic continues to be determined as the field rapidly evolves. When it comes to the role of medical physicists in conjunction with AI, an open discussion of the exact responsibilities is still ongoing, and feedback is encouraged from all the members of the community. So, what can medical physicists do? They can use AI to enhance quality improvement and safety by analyzing medical data to identify trends, patterns, and outliers.^9^ This can lead to the identification of areas for improvement or potential safety hazards and help them enter the realm of Responsible AI. AI can also improve diagnostic and therapeutic techniques by enhancing the quality of medical imaging and automating image interpretation.^10^ Furthermore, AI can help in integrating diagnostics, personalized medicine, and theragnostics by analyzing large datasets to tailor treatment plans to individual patients.^11^ This can lead to more effective and personalized care. AI can also automate routine tasks in medical physics, such as treatment planning and QA processes, leading to increased efficiency.^12^ Lastly, AI techniques like machine learning and deep learning can be leveraged for research and development to analyze complex datasets, discover patterns, and develop innovative techniques for disease detection, treatment, and monitoring.^13^

Whether it involves developing AI‐driven solutions like automated segmentation, dose calculations, addressing intricate problems in the clinic, or potentially even contributing to open‐source AI initiatives, such activities will empower medical physicists to enhance their skills and make tangible contributions to the advancement of healthcare. Embracing AI not only fosters a sense of accomplishment but also opens doors to the world of `automation' and scaling that will pervade all technologies of the future. The AHAIBC committee is at the center of bringing the medical physicist forward by developing curriculum concepts, bootcamps, and engendering engagement for our society.

Integration of AI into the realm of medical physics education is critical, especially considering the potential significance of incorrect AI usage or misapplication. The physicist is responsible for installing and commissioning the AI software, ensuring the modeling is not biased, performing continuing QA on the hospital data and processes, and establishing efficient resource management. Embracing education in AI offers new benefits for medical physicists as it is already revolutionizing various industries and professional practices and we need to be equally prepared.

One way to engage and prepare healthcare professionals for the upcoming AI wave is to start with the roots of quality safety and assurance. To do this, we should enable a comprehensive QA program that encompasses all clinical operations related to medical fields including radiology, nuclear medicine, and radiation oncology. Ensuring the safe operation of hardware, software, clinical operation processes and machinery is of utmost importance and one of the most crucial responsibilities of a medical physicist. A Real AI approach can be highly beneficial in achieving the goal of safe clinical implementation.

## 2 CONCLUSIONS

Understanding the potential and limitations of AI serves as a cornerstone for fostering engagement not only within our profession but with other healthcare providers. Continuous learning and participation in hands‐on experience are essential components for navigating the complexities of AI applications within healthcare. Collaboration, networking, and exploring AI's purpose and impact are equally vital in this journey. Additionally, some physicists may choose personal projects, embracing challenges in small groups, and actively contributing to AI‐focused teams to amplify the motivation and expertise of our field. Insights through personal and collaborative opportunities ultimately provide for and encourage professional growth and innovation within our medical physics field. Some medical physicists may be able to attend specialty meetings and conferences dedicated to AI which further enriches their knowledge base and provides them avenues for fruitful collaboration. There are successful educational programs such as the Radiological Society of North America Artificial Intelligence (RSNA AI)‐certificate program.^14^ Interdisciplinary cooperation and inter‐institutional collaboration for AI experts is of paramount importance for integrating AI into medical physicists' practice on a larger scale, and mechanisms enabling this collaboration should be provided to the community.

In summary, the authors believe that being prepared for and embracing the changes that AI is already bringing at the current time will benefit our community, healthcare, patient care, and society at large immediately and for the future. We are at a critical juncture, which can be considered a fourth industrial revolution, where AI and automation are applied more broadly. Medical physicists have a pivotal role to play in this revolution. We need to position ourselves at the forefront of ‘Real AI’ and lead the charge in this exciting new era. It is time for action, and we can take the first steps with potentially just a few ABCs.

## AUTHOR CONTRIBUTIONS

All authors contributed their efforts in writing and editing this call for action. ChatGPT search engine has been utilized to provide additional background to the subject of matter for illustrative purposes.

## CONFLICT OF INTEREST STATEMENT

The authors declare no conflicts of interest.

## LARGE LANGUAGE MODEL AI TOOL USE DISCLOSURE

The content for this call for action has been edited with the help of large language models ChatGPT and Google NotebookLM.
